# Cordycepin and N^6^-(2-Hydroxyethyl)-Adenosine from *Cordyceps pruinosa* and Their Interaction with Human Serum Albumin

**DOI:** 10.1371/journal.pone.0121669

**Published:** 2015-03-26

**Authors:** Zebin Meng, Jichuan Kang, Tingchi Wen, Bangxing Lei, Kevin David Hyde

**Affiliations:** 1 The Engineering and Research Center for Southwest Bio-Pharmaceutical Resources of National Education Ministry of China, Guizhou University, Huaxi, Guiyang, Guizhou Province, PR China; 2 Guizhou Bioresource Development and Utilization Key Laboratory, Guizhou Normal College, Guiyang, Guizhou Province, China; 3 Institute of Excellence in Fungal Research, School of Science, Mae Fah Luang University, Chiang Rai, Thailand; Islamic Azad University-Mashhad Branch, Mashhad, Iran, IRAN, ISLAMIC REPUBLIC OF

## Abstract

*Cordyceps pruinosa* (CP) is often used as Traditional Chinese Medicine, but the substance basis of its medicinal properties is unclear. In this study, two compounds were isolated from CP cultures by column chromatography, and identified as cordycepin and N^6^-(2-hydroxyethyl)-adenosine (HEA) by Nuclear Magnetic Resonance. In order to understand the efficacy of these two substances as potential therapeutic agents, it is necessary to explore their binding with proteins. The molecular mechanisms of interaction between cordycepin, HEA and human serum albumin (HSA) were studied using UV and fluorescence spectroscopy. The bingding constants between HSA and cordycepin were 4.227, 3.573 and 3.076 × 10^3^·at 17, 27 and 37°C respectively, and that of HSA and HEA were 27.102, 19.409 and 13.002 × 10^3^·at the three tempretures respectively. Both cordycepin and HEA can quench the intrinsic fluorescence of HSA via static quenching, and they can bind with HSA to form complexes with a single binding site. The interaction forces between cordycepin and HSA were determined as electrostatic and hydrophobic, and those of HEA and HSA were hydrogen bonding and van der Waals forces. Using Foster's equation, the distance between fluorophores of cordycepin and HSA, and HEA and HSA are estimated to be 5.31 nm and 4.98 nm, respectively. In this study, cordycepin was isolated for the first time from CP, and will provide a new source of cordycepin and expand the use of this taxon. The interaction mechanisms between cordycepin and HSA was studied for the first time, which will provide a useful guide for the clinical application of cordycepin. The pharmacological importance of this study is to understand the interaction of HSA with cordycepin and HEA, which will be essential for the future designing of drugs based on the two compounds.

## Introduction

*Cordyceps sensu lato* is one of the most important fungal groups of invertebrate pathogens with about 530 species (Index Fungorum 2014) [1]. Searching for bioactive compounds from *Cordyceps sensu lato* is an important way to screen for new medicines. *Cordyceps pruinosa* Petch (CP) belongs to *Cordyceps sensu stricto* and has potential medicinal application [2–3]. Polysaccharides isolated from CP have been shown to improve cellular immune functioning [4]. Methanol extracts of CP inhibited inflammation [5–6] while the butanol fractions of it induced apoptosis in HeLa cells [7]. Although CP showed a series of bioactivities, these were only established via crude extracts but not pure compounds, thus the substance basis of these bioactivities remains unclear.

**Fig 1:**
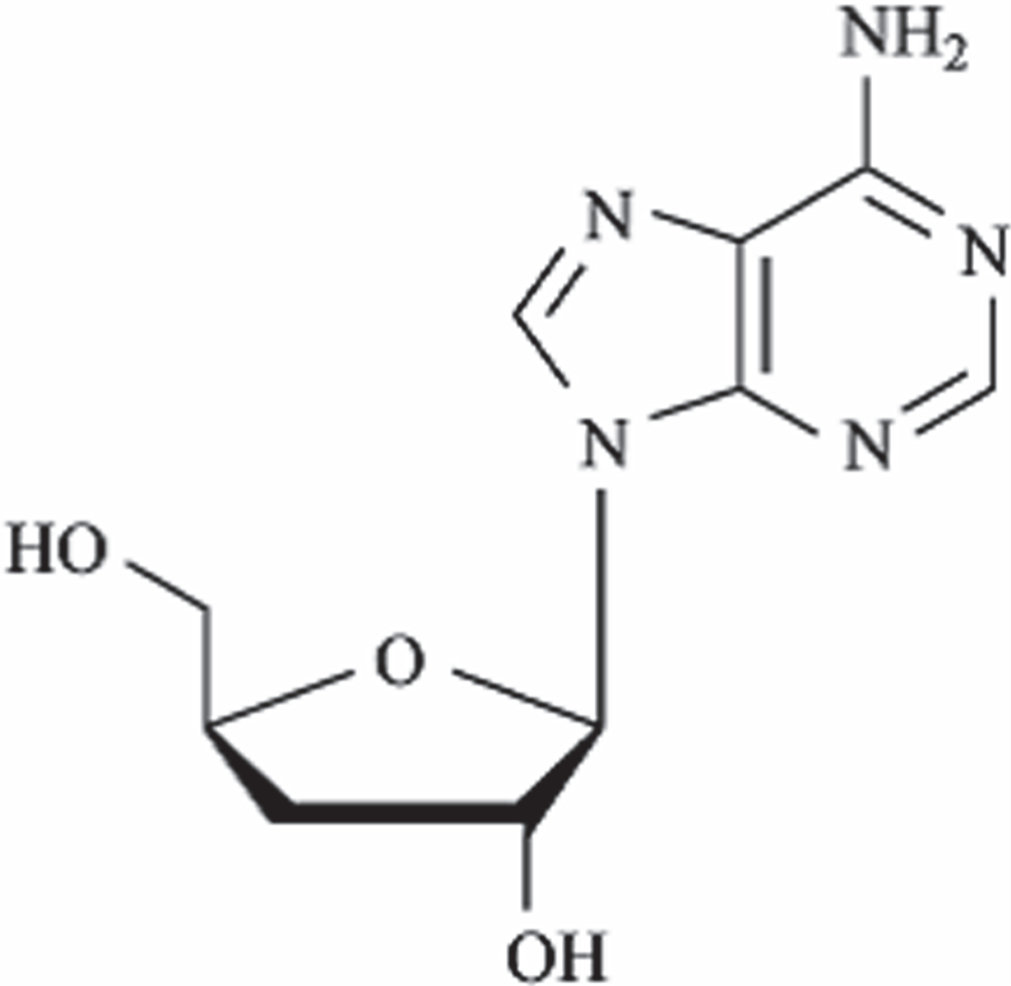
Chemical structure of cordycepin.

Cordycepin (3'-deoxyadenosine) is the major bioactive component from *Cordyceps militaris* that has been widely used as a Traditional Medicine and healthy food in oriental countries. Cordycepin is an adenosine derivative with the formula of C_10_H_13_N_5_O_3_, molecular weight 251 D and the chemical structural shown in Fig. 1. Since 1960s, broad pharmacological functions of cordycepin have been discovered, which include: anti-tumor [8], anti-bacterial [9], anti-virus [10], immunomodulatory [11], anti-inflammatory [12], hyperlipemia regulation [13], anti-aging [14], neuroprotective [15], promoting learning and memory [16], anti-oxidant activity [17], apoptosis [18] and has a positive effect on rheumatoid arthritis [19]. In the last decade, studies targeting cordycepin as a therapeutic agent have been in progress, especially application to leukemia (*ClinicalTrials*.*gov*, verified by OncoVista, Inc., 2009) [20–21]. In addition, preclinical assessment of cordycepin and deoxycoformycin in the treatment of African trypanosomiasis in second-stage has been tested [22].

**Fig 2:**
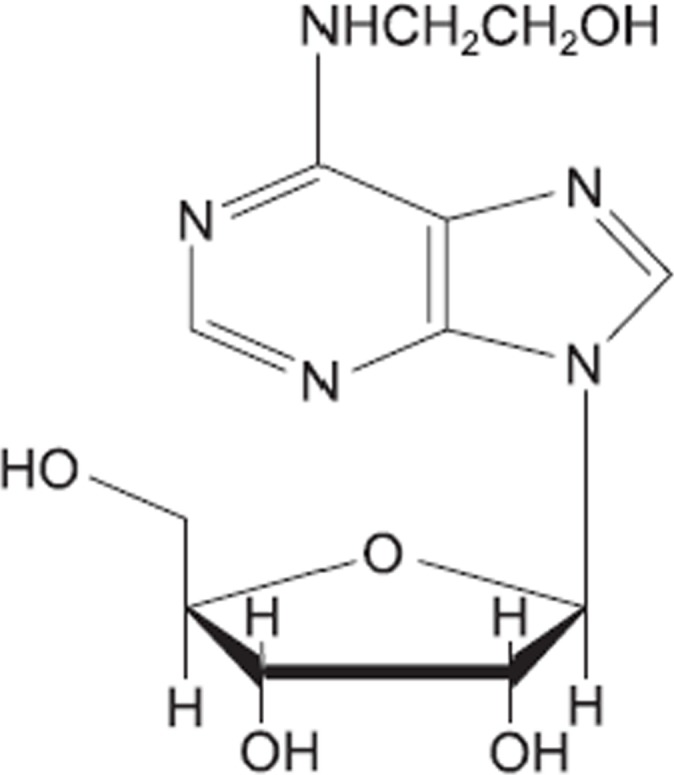
Chemical structure of N^6^-(2-hydroxyethyl)-adenosine.

One of the main bioactive compounds produced by CP is N^6^-(2-hydroxyethyl)-adenosine (HEA) (C_12_H_17_N_5_O_5_, Fig. 2). The compound behaves as a Ca^2+^ antagonist, an inotropic agent, has radiation resistance [23], causes hypomobility in mice [24], is an analgesic [25], protects the brain [26] and has anti-tumor properties [27]. In addition, HEA can bind with human serum albumin (HSA) to form a complex by hydrophobic interaction [28].

HSA is the most abundant protein in the plasma, which plays important role in transporting, distribution, storing and metabolism of many exogenous ligands, such as drugs, fatty acids, and amino acids. It is a globular protein composed of 585 amino acids, which can bind with different drug types and various small bioactive molecules, such as metal cations and fatty acids. As a consequence, studies on the interaction between HSA and drugs can offer information of drug action to understand the distribution and absorption of the drugs [29–33].

Although there are about 530 species in *Cordyceps sensu lato*, cordycepin had only been reported in 12 species [34]. So far there was no report on cordycepin isolation from CP as well as no report concerning the interaction of cordycepin with HSA. HEA was first found in CP and few reports concerning its interaction with HSA are available. In the present work, the two compounds of adenosine derivatives, cordycepin and HEA, were isolated from CP, and their interaction with HSA were studied systematically under simulated physiological conditions. The binding mechanism and the thermodynamic parameters were characterized by fluorescence approach and UV absorption spectroscopic assay.

## Materials and Methods

### Reagents and Buffers

All chemicals were of the reagent grade. All buffers were prepared using deionized water from a Milli-Q water purification system (Millipore Corp., USA) with a resistivity >18.2 MQ·cm. The pH values were measured with an ORION pH meter and a 0079 microelectrode (ORION, USA), respectively, at 25°C.

### Strain and Fermentation

The CP strain (GZUCC 8552) used in this study is deposited in Guizhou University Culture Collection, Guizhou Province, China. The strain was activated on PDA and then transferred to the seed culture by punching out 10 mm of the agar disc with a sterilized self-designed cutter. The seed culture was placed in a 250 mL flask containing 100 mL of basic medium (sucrose 10 g/L, glucose 5 g/L, glycerol 10 g/L, soybean meal 5 g/L, yeast extract 1 g/L, KH_2_PO_4_·3H_2_O 1 g/L, MgSO_4_·7H_2_O 10 g/L, KCl 0.5 g/L, FeSO4·7H_2_O 0.01 g/L with 1000 mL distilled water), on a rotary shaking incubator at 26°C in darkness with 100 rev/min for 4 d. The liquid static medium was prepared by mixing 200 mL of basic medium (sucrose 10 g/L, glucose 10 g/L, peptone 10 g/L, MgSO_4_·7H_2_O 1 g/L, K_2_HPO_4_ 1 g/L and KH_2_PO_4_·3H_2_O 0.5 g/L with 1000 mL distilled water) in a cylindrical glass bottle. The media were autoclaved for 30 min at 121°C and each bottle was inoculated with 5 mL liquid inoculum of the seed culture. All bottles were incubated at 26°C in darkness for 120 days via liquid static culture. All the mycelia were harvested and dried to a constant weight at 55°C.

### Isolation of Cordycepin and HEA

The CP mycelia powder (1 kg) were extracted with 75% (v/v) ethanol (10 L × 3) for three times in 30 min at room temperature using an ultrasonic cleaner (Ningbo Scientz Boitechnology, China), and then the combined extracts were concentrated to 2 L under reduced pressure. The concentrated extracts was loaded on a macroporous resin AB-8 chromatography with a gradient elution of ethanol/water (0%, 10%, 30%) to yield three fractions. The 30% fraction was choosen for further isolation via HPLC (Agilent 1100 series, USA) analysis. HPLC was with an RP-C18 column (5 μm, 4.6 × 150 mm) (Upelco, Bellefonte, PA, USA). The mobile phase consisted of water and methanol (90:10, v/v). Elution was performed at a flow rate of 1 mL min^–1^ with the column temperature at 45°C, injection volume of 10 μL and the UV wavelength of 254 nm [35].

### Preparative reversed-phase HPLC

The 30% fraction of CP was filtered through a 0.45 μm pore-size Millex-HV hydrophilic PVDF syringe filter (Millipore) for further experiment. Preparative reversed-phase HPLC separation of 30% fraction was performed on the Agilent 1100 series HPLC (Agilent Technologies, USA) equipped with an additional fraction collector. Separation took place on a ZORBAX SB-C18 (21.2 mm × 250 mm; 5 μm particle size; 110 Å pore size) column. The mobile phases of water and methanol (v:v, 90:10) were used. The flow rate and column temperature were maintained at 8 mL/min and 30°C respectively, and 100 μL injection repeatedly. The fractions were collected using automatic peak detection. Two compounds were isolated after the pooled fractions were rotary evaporated under vacuum and freeze-dried.

### General experimental procedures

Melting point was measured with an XT-4 apparatus and uncorrected. The ^1^H and ^13^C NMR spectra were recorded on an INOVA-400 (Varian, San Francisco, USA) instrument using TMS as internal standard. ESI-MS was measured with a HP-5973 mass spectrometer. EIMS measurements were undertaken on a VG Autospec-3000 mass spectrometer (VG, Manchester, UK).

### UV Absorption Spectra Assay

The UV absorption spectra of HSA were detected by UV spectrophotometry (UV-2450PC, Shimadzu). The spectra of 0.5 μM HSA (Sigma company) were analyzed with Tris-HCl buffer (pH 7.4) as the control. Different concentrations of cordycepin (0, 10, 20, 30, 40 and 50 μM) were added into the Tris-HCl buffer. The sample was incubated for 30 min before assaying. The spectra of cordycepin, HSA, and mixture of cordycepin and HSA were recorded, respectively. In the same way, different concentrations of HEA (0, 10, 20, 30, 40 and 50 μM) were added into the Tris-HCl buffer and the buffer containing 0.5 μM HSA. The spectra of HEA, HSA, and mixture of HEA and HSA were also recorded.

### Fluorescence Spectra Assay

The fluorescence spectra were measured with a Cary Eclipse Fluorescence Spectrofluorometer (Agilent Technologies, USA) equipped with a single cell peltier accessory. The fluorescence spectra of HSA were detected in Tris-HCl buffer (pH 7.4) when the width of entrance and exit slit was 5 nm and the scanning speed was 750 nm•sec^−1^. One μM HSA was mixed with different concentrations of cordycepin (0, 10, 20, 30, 40 and 50 μM) in the Tris-HCl buffer (a total accumulated volume of 2000 μL, pH 7.4). The reactions were assayed at 17, 27 and 37°C. In the same way, 1 μM HSA was mixed with different concentrations of HEA (0, 10, 20, 30, 40 and 50 μM). The type of fluorescent quenching was determined following the Equation (1) as previously described [36].

$${}_{{\text{F}}_{\text{0}}\text{/F}=\text{1}+{\text{K}}_{\text{q}}\tau_{0}\left[Q\right]=1+K_{sv}[Q]}$$ (1)

Where *F*_*0*_ is the fluorescence intensity in the absence of quencher, *F* is the fluorescence intensity in the presence of quencher concentration, *K*_*q*_ *τ*_*0*_ is the quenching rate constant of biomacromolecule, *K*_*sv*_ is the quenching rate constant of dynamic quenching and *[Q]* is fluorescence lifetime of biomacromolecule without quencher. *τ*_*0*_ is usually about 10^–8^ sec [36–37]. The maximal collisional quenching rate constant was 2×10^10^ L·mol^−1^·s^−1^ for all classes of the biomolecule [38].

The binding constant and the number of binding sites were detected following Equation (2) as previously described [39–40].

$$\mathrm{lg}((F_{0}-F)/F)=\mathrm{lg}K+n\mathrm{lg}[Q]$$ (2)

Where *K* is the binding constant and *n* is the number of binding sites.

The acting forces include hydrogen bonds, Van der Waals forces, electrostatic forces, and hydrophobic interaction forces between small molecule drugs and biomacromolecules. The reaction enthalpy change is regarded as a constant when the temperature hardly changes. Thus, the types of acting forces can be determined following Equations (3 and 4) as previously described [41].

$$\ln K=-\frac{\text{Δ}H}{RT}+\frac{\text{Δ}S}{R}$$ (3)

ΔG=ΔH−TΔS (4)

Where *K* is the binding constant, *T* is the temperature, *R* is the gas molecule constant, *ΔH* is the binding enthalpy change, *ΔS* is the binding entropy change, *ΔG* is the Gibbs’ free energy change.

The acting force were hydrophobic interaction force, hydrogen bonds and Vander Waals force, and electrostatic force, respectively, when *ΔH*>0 and *ΔS*>0, *ΔH*<O and *ΔS*<0, and *ΔH*≈0 and *Δ*S>0 [37].

According to the Forster non-radioactive energy transfer theory (Equations (5)–(7)) [37–38], the energy-transfer effect is related not only to the distance between acceptor and donor (*r*), but also to the critical energy transfer distance (with a transfer efficiency of 50%, *R*_*0*_).

$$E=R_{0}^{6}/(R_{0}^{6}+r^{6})$$ (5)

$$E=1-F/F_{0}$$ (6)

$$R_{0}^{6}=8.8\times 10^{-25}K^{2}N^{-4}\phi_{P}J$$ (7)

Where *K* ^*2*^ is the spatial-orientation factor of dipole, *N* is the refractive index of medium, *Φ* is the fluorescence quantum yield of donor, *E* is the energy transfer effect, *F* is the fluorescence intensity when C_(Drugs)_/C_(HSA)_ = 1:1. In this study, *K* ^*2*^ = 2/3, *N* = 1.336, and *Φ* = 0.118. *J* is the overlap integral of the fluorescence emission spectrum of donor and the absorption spectrum of acceptor when C_(Drugs)_/C_(HSA)_ = 1:1 (Equation (8)).

$$J=\left(\sum I_{P}(\lambda )\varepsilon_{D}(\lambda )\lambda^{4}\text{Δ}\lambda \right)/(\sum I_{P}(\lambda )\text{Δ}\lambda )$$ (8)

Where *I*_*p*_ *(λ)* is the fluorescence intensity of fluorescent donor at wavelength *λ*, and *ε*_*D*_ *(λ)* is the molar absorbance of acceptor at wavelength *λ*.

### Statistical Analysis

The data were analyzed by F-test and SPSS 13.0 (SPSS 13.0, 2005, SPSS Inc., Chicago, USA) according to the general linear model. *P*<0.05 was considered significant and *P*<0.01 was considered extremely significant. The data are expressed as mean ± SD. The graph were made by OriginLab OriginPro 8.5 (OriginLab Corporation, USA) and Excel 2010 (Microsoft Corporation, USA)

## Results

### Two compounds isolated from CP

Two compounds, compound 1 and compound 2, were isolated by column chromatography. The yield of compound 1 and compound 2 were 46 and 23.7 mg, respectively. Their physical and chemical characteristics were as follows.

### Compound 1 white powder (MeOH)

mp 227 ~ 228°C. ESI-MS m/z 252 [M + H]^+^. ^1^H-NMR (DMSO-*d*_6_, 600 MHz) δ: 8.13 (1H, s, H-2), 8.34 (1H, s, H-8), 5.84 (1H, d, *J* = 2.4 Hz, H-1′), 4.55 (1H, m, H-2′), 2.22 (1H, ddd, *J* = 13.3, 8.6, 5.9 Hz, Ha-3′), 1.90 (1H, ddd, J = 13.2, 6.5, 3.4 Hz, Hb-3′), 4.34 (1H, m, H-4′), 3.65 (1H, ddd, *J* = 12.0, 5.5, 3.3 Hz, Ha-5′), 3.51 (1H, ddd, *J* = 12.0, 5.5, 4.1 Hz, Hb-5′), 5.78 (1H, d, *J* = 4.3 Hz, 2′-OH), 5.29 (1H, t, *J* = 5.7 Hz, 5′-OH), 7.26 (2H, brs, N_6_H_2_). ^13^C-NMR (DMSO-*d*_6_, 125 MHz) δ: 152.7 (C-2), 149.0 (C-4), 119.2 (C-5), 156.2 (C-6), 139.5 (C-8), 91.1 (C-1′), 74.9 (C-2′), 34.2 (C-3′), 81.0 (C-4′), 62.8 (C-5′). All these data were consistent with those of cordycepin [42].

### Compound 2 white powder (MeOH)

mp 194 ~ 196°C. ESI-MS m/z 312 [M + H]^+^. ^1^H-NMR (DMSO-*d*_6_, 400 MHz) δ: 8.19 (1H, s, H-2), 8.32 (1H, s, H-8), 5.85 (1H, d, *J* = 6.4 Hz, H-1′), 4.57 (1H, dd, *J* = 11.3, 6.0 Hz, H-2′), 4.12 (1H, m, H-3′), 3.96 (1H, dd, J = 6.5, 3.1 Hz, H-4′), 3.67 (1H, dt, *J* = 12.1, 3.8 Hz, H-5′), 3.56 (2H, m, H-1"), 3.56 (2H, m, H-2"), 5.44 (1H, d, *J* = 6.3 Hz, 2′-OH), 5.26 (1H, d, *J* = 4.65 Hz, 3′-OH), 5.42 (1H, dd, *J* = 7.1, 4.5 Hz, 5′-OH), 7.66 (1H, brs, N^6^H), 4.90 (1H, brs, 2"-OH). ^13^C-NMR (DMSO-*d*_6_, 400 MHz) δ: 152.7 (C-2), 148.5 (C-4), 120.1 (C-5), 154.5 (C-6), 140.2 (C-8), 88.3 (C-1′), 73.9 (C-2′), 70.9 (C-3′), 86.3 (C-4′), 62.0 (C-5′), 42.8 (C-1"), 60.0 (C-2"). All these data were consistent with those of N^6^-(2-hydroxyethyl)-adenosine [27].

### UV absorption spectra

**Fig 3:**
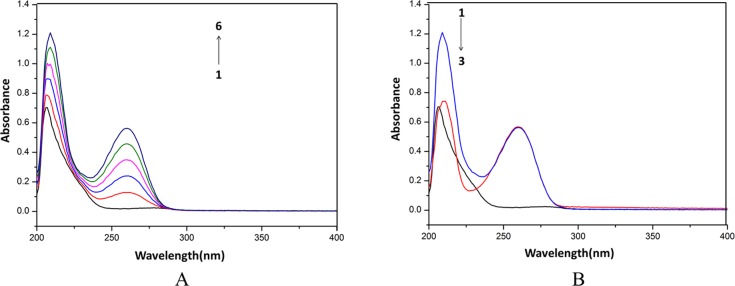
Effects of cordycepin on HSA by UV spectra. A: The concentration of HSA was 0.5 μM while the cordycepin concentration corresponding to 0, 10, 20, 30, 40 and 50 μM from (1 to 6). B: 1, 0.5 μM HSA + 50 μM cordycepin; 2, 50 μM cordycepin; 3, 0.5 μM HSA.

**Fig 4:**
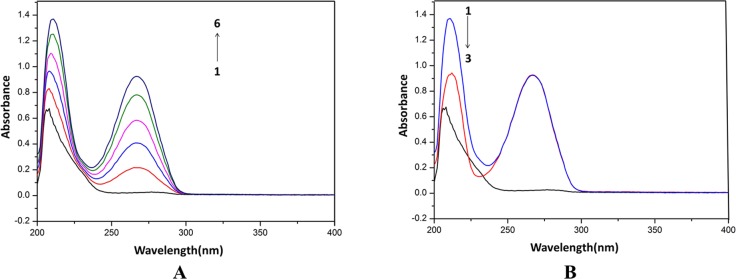
Effects of HEA on HSA by UV spectra. A: The concentration of HSA was 0.5 μM while the HEA concentration corresponded to 0, 10, 20, 30, 40 and 50 μM from (1 to 6). B: 1, 0.5 μM HSA + 50 μM HEA; 2, 50 μM HEA; 3, 0.5 μM HSA.

UV–vis absorption measurement is usually used to explore the structural change and compound formation. The UV–vis absorption spectra at about 210 nm represents the α-helical structure of HSA [43]. The effect of cordycepin on UV absorption spectra of HSA is shown in Fig. 3, and that of HEA shown in Fig. 4. The absorbance (208 nm) intensity of HSA increased with the increasing concentration of cordycepin, with an apparent red shift from 208 to 211 nm. The absorbance (206 nm) intensity of HSA increased with the increasing concentration of HEA, with an apparent red shift from 206 to 209 nm.

The shift at 268 nm was not-significant (*P*<0.05) at the tested concentrations of cordycepin. The increase and red shift of the absorption indicated that the bind of the chemical induced the loosening and unfolding of the protein skeleton and decreased the hydrophobicity of the micro-environment of the aromatic amino acid residue. The UV spectra of cordycepin, HSA and their mixture showed different maximum absorption peaks, suggesting that cordycepin form a HSA-cordycepin complex via reacting with HSA. However, the maximum absorption varied from 208 to 211 nm, demonstrating that cordycepin could affect the conformation of HSA.

The absorbance (206 nm) intensity of HSA increased with an increasing concentration of HEA, with an apparent red shift from 206 to 209 nm. The shift at 260 nm was not-significant (*P*<0.05) at the tested concentrations of HEA. The increase and red shift of the absorption indicated that the bind of HEA induced the loosening and unfolding of the HSA skeleton and decreased the hydrophobicity of the micro-environment of the aromatic amino acid residue. The UV spectra of HEA, HSA and their mixture showed different maximum absorption peaks, indicating that HEA form a HSA-HEA complex via reacting with HSA.

### Fluorescence quenching of HSA by drugs

**Fig 5:**
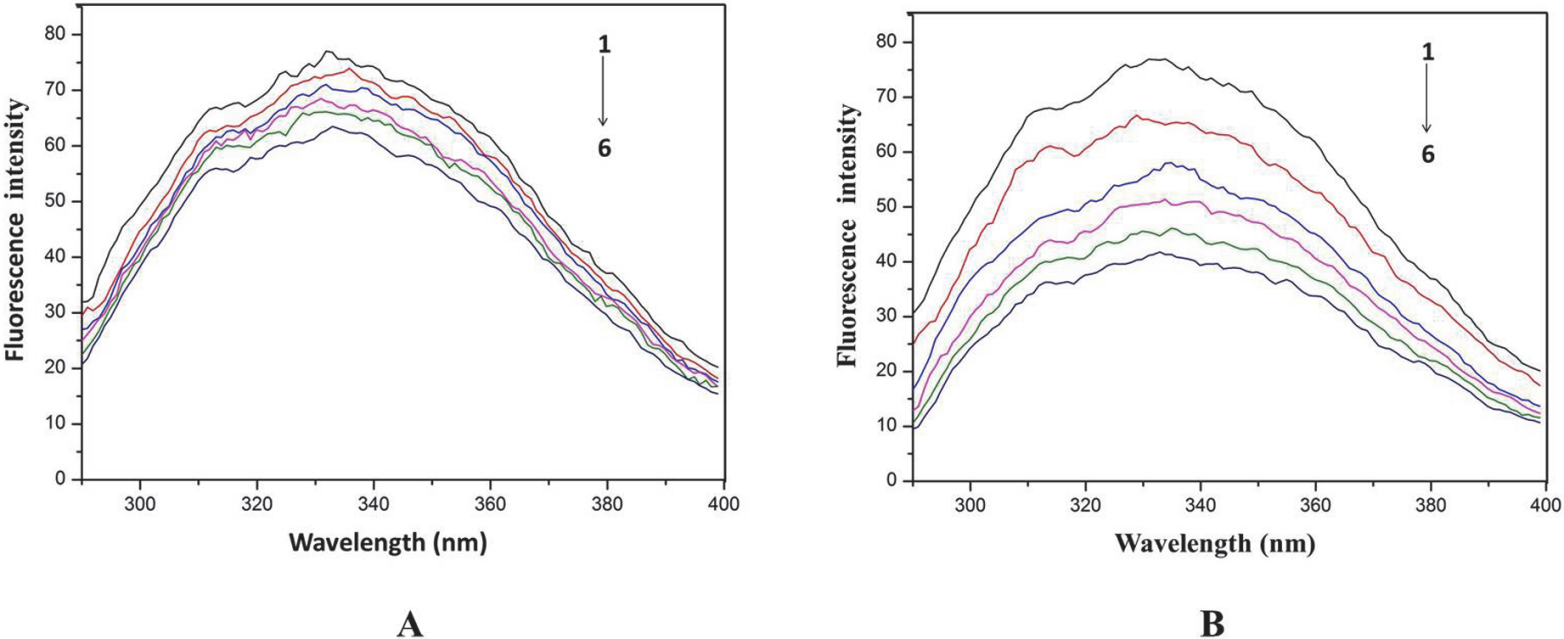
Effect of drug on fluorescence spectra of HSA. A: Effect of cordycepin on fluorescence spectra of HSA; the concentration of HSA was 1 μM while the cordycepin concentration corresponded to 0, 10, 20, 30, 40 and 50 μM (from 1 to 6). B: Effect of HEA on fluorescence spectra of HSA. The concentration of HSA was 1 μM while the HEA concentration corresponding to 0, 10, 20, 30, 40 and 50 μM (from 1 to 6).

The effect of cordycepin on fluorescence spectra of HSA is shown in Fig. 5A, and that of HEA in Fig. 5B. The excitation and emission wavelengths were 280 nm and 330 nm, respectively. The results showed that the fluorescence intensity of HSA decreased with the increasing concentrations of cordycepin and HEA, respectively, indicating that both cordycepin and HEA can quench the intrinsic fluorescence of HSA.

**Fig 6:**
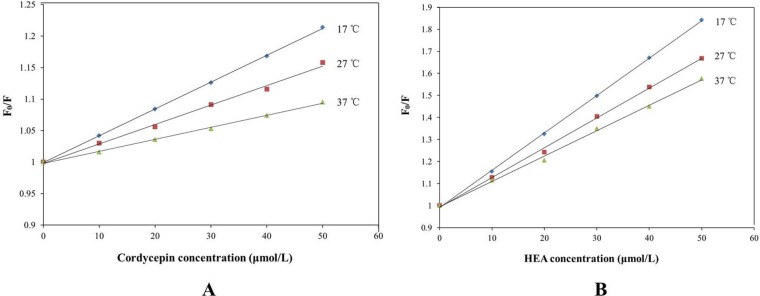
A: Stern–Volmer plot for cordycepin–HSA at different temperatures; B: Stern–Volmer plot for HEA–HSA at different temperatures.

The quenching rate constant of biomacromolecule *K*_*q*_ was calculated using Equation 1. The *K*_*q*_ values for cordycepin were 4.257×10^11^, 3.094×10^11^ and 1.903×10^11^ L·mol^−1^·s^−1^ at 17, 27 and 37°C, respectively (Table 1 and Fig. 6A). The *K*_*q*_ values for HEA at 17, 27 and 37°C were 1.695×10^12^, 1.352×10^12^ and 1.151×10^12^ L·mol^−1^·s^−1^, respectively (Table 1 and Fig. 6B). The *K*_*q*_ values of HSA-cordycepin and HSA-HEA decreased with increasing temperature, and these *K*_*q*_ values of HSA-cordycepin and HSA-HEA were all far greater than the maximal collisional quenching rate constant (2×10^10^ L·mol^−1^·s^−1^) of all classes of the biomolecule, which suggested that both fluorescent quenchings between HSA and cordycepin, and HSA and HEA were caused by static quenching rather than by dynamic collisions.

**Table 1:** Stern-Volmer equations and quenching constants for fluorescence quenching of HSA by cordycepin and HEA at different temperatures.

Quenching agent	T/K	F_0_/F~[Q] equation	*K*_*q*_ (×10^11^ ·L·mol^−1^·s^−1^)	*R* ^*2*^
cordycepin	290	y = 4.257×10^3^x +0.9992	4.257±0.070	0.9998
	300	y = 3.094×10^3^x +0.9978	3.094±0.076	0.9952
	310	y = 1.903×10^3^x +0.9981	1.903±0.059	0.9980
	290	y = 1.695×10^4^x +0.9912	16.95±0.277	0.9997
HEA	300	y = 1.352×10^4^x +0.9923	13.52±0.332	0.9984
	310	y = 1.151×10^4^x +0.9948	11.51±0.430	0.9974

**Fig 7:**
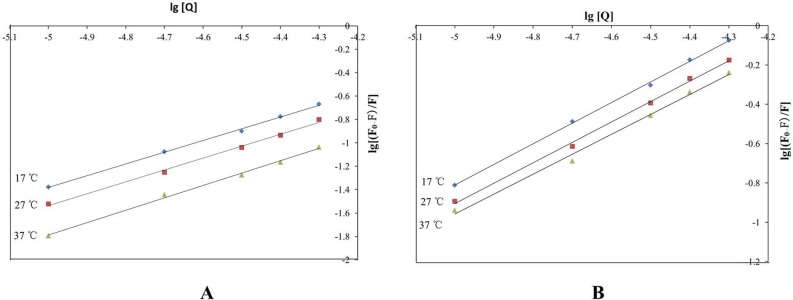
Plot of log [(F_0_-F)/F] vs log [Q] at different temperatures of cordycepin and has (A); Plot of log [(F_0_-F)/F] vs log [Q] at different temperatures of HEA and has (B).

The binding constant (*K*) and the number of binding sites (*n*) were calculated following Equation 2. The *K* values between HSA and cordycepin were 4.227 ×10^3^, 3.573×10^3^, 3.076×10^3^ at 17, 27 and 37°C, respectively (Table 2 and Fig. 7A), indicating that there was a strong binding force between HSA and cordycepin. The *n* values were 1.0012, 1.0181 and 1.0594 at 17, 27 and 37°C, respectively, suggesting that HSA and cordycepin formed a HSA-cordycepin complex at one binding site.

**Table 2:** *K* values of HSA at different temperatures.

Quenching agent	T/K	lg((F_0_-F)/F)~ lg[Q]	*K* (×10^3^)	*n*	*R* ^*2*^
cordycepin	290	y = 1.0012x +3.626	4.227±0.083	1.0012	0.9982
	300	y = 1.0181x +3.553	3.573±0.193	1.0181	0.9957
	310	y = 1.0549x +3.488	3.076±0.085	1.0549	0.9960
	290	y = 1.0488x +4.433	27.102±0.272	1.0488	0.9988
HEA	300	y = 1.0386x +4.288	19.409±0.649	1.0386	0.9976
	310	y = 1.0144x+4.114	13.002±0.310	1.0144	0.9945

The *K* values between HSA and HEA were 27.102×10^3^, 19.409×10^3^, 13.002×10^3^ at 17, 27 and 37°C, respectively (Table 2 and Fig. 7B), the *n* values were 1.0488, 1.0386 and 1.0144 at 17, 27 and 37°C, respectively. All these data suggested that HSA and HEA formed a HSA-HEA complex at one binding site with a strong binding force.

### Thermodynamic analysis and the nature of the binding forces

**Fig 8:**
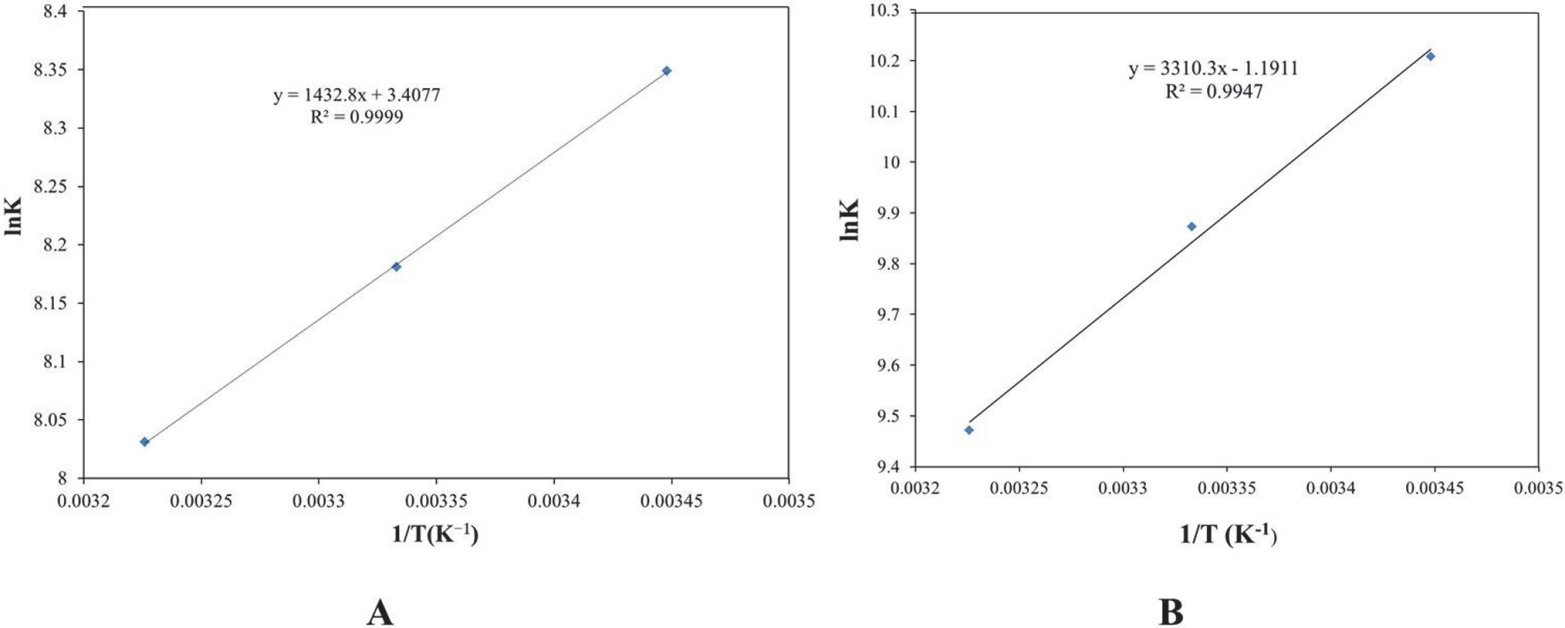
*ΔH*, *ΔG*, and *ΔS* of the reaction between cordycepin and has (A). *ΔH*, *ΔG*, and *ΔS* of the reaction between HEA and has (B).

The *ΔH*, *ΔG*, and *ΔS* of the reaction between HSA and various drugs were calculated using Equation (2) and (4) (Table 3 and Fig. 8). The *ΔG* of HSA and cordycepin at 17, 27 and 37°C were −20.128, −20.412, −20.695 kJ·mol^−1^, respectively and all the figures were smaller than zero, indicating that the reaction between HSA and cordycepin occurs spontaneously. The *ΔH* = −11.912 kJ/mol and *ΔS* = 28.332 J/mol•k (*ΔH*<0, *ΔS*>0) suggested that the interacting forces were electrostatic and hydrophobic.

**Table 3:** *ΔH*, *ΔG*, and *ΔS* of the interaction between cordycepin and HSA, HEA and HSA at different temperatures.

Quenching agent	T/K	*K* (×10^3^)	*ΔG* (kJ·mol^−1^)	*ΔH* (kJ·mol^−1^)	*ΔS* (J·mol^−1^·K^−1^)
cordycepin	290	4.227±0.083	−20.128	−11.912±0.086	28.332±0.570
	300	3.573±0.193	−20.412		
	310	3.076±0.085	−20.695		
	290	27.102±0.272	−24.650		
HEA	300	19.409±0.649	−24.551	−27.522±0.410	−9.903±0.639
	310	13.002±0.310	−24.452		

The *ΔG* of HSA and HEA at 17, 27 and 37°C were −24.650, −24.551 and −24.452 kJ·mol^−1^ respectively, which indicated that the reaction between HSA and HEA occurs spontaneously. The *ΔH* = −27.552 kJ/mol and *ΔS* = −9.903 J/mol•k (*ΔH*<0, *ΔS*<0) demonstrated that the interaction forces are hydrogen bonding and van der Waals force.

Though HEA and cordycepin are structurally similar, there are some differences in their structure. HEA has a more hydroxy at the ribose than that of cordycepin, and has a more hydroxyethyl at purine than that of cordycepin, which make their spatial structures different. And the two groups have “O”, which provide HEA having more chances to establish hydrogen with HSA. And the different structure may make different binding site with HSA. So the binding affinity of HEA is greater than cordycepin.

### Energy transfer from HSA to drugs

**Fig 9:**
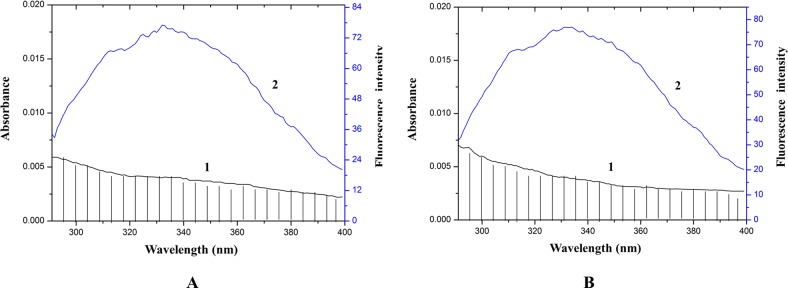
Stern-Volmer equations and quenching constants for fluorescence quenching of HSA by cordycepin and HEA at different temperatures. C_(cordycepin)_ = C _(HSA)_ = C _(HEA)_ = 1 μM.

The overlap between the drug absorption spectrum and the HSA fluorescence emission spectrum is shown in Fig. 9. The overlap integral (*J*) could be assessed by integration with the Metlab program using Equation 8. According to the Forster non-radioactive energy transfer theory (Equations 5–7), the distance between acceptor and donor (*r*) and the critical energy transfer distance (with a transfer efficiency of 50%, *R*_*0*_) were calculated.

The *J* of HSA and cordycepin was 0.41213×10^–13^ mol^−1^·cm^−1^·nm^4^, *E* was 0.02594 at 17°C, *R*_*0*_ and *r* were 2.90 nm and 5.31 nm respectively. The *J* of HSA and HEA was 0.42811×10^–13^ mol^−1^·cm^−1^·nm^4^, *E* was 0.03896 at 17°C, *R*_*0*_ and *r* were 2.92 nm and 4.98 nm respectively. It was reported that non-radioactive energy transfer occurs between the donor and receptor when the distance between two molecules was smaller than 7 nm [29]. In the present study, the *r* was smaller than 7 nm at all reaction temperatures, suggesting that non-radioactive energy transfer quenched fluorescence.

## Discussion

In this study, two nucleoside analogues compounds, cordycepin and HEA were isolated from CP by column chromatography and identified by NMR. The effect of cordycepin and HEA on biological activity and conformation of HSA were studied, and their interaction mechanisms were tested by fluorescence and UV spectroscopy. The results indicated that both cordycepin and HEA quenched the intrinsic fluorescence of HSA via static quenching, and they bound with HSA to form a complexe with one binding site. The interacting forces between cordycepin and HSA were determined as electrostatic and hydrophobic, however those of HEA and HSA were hydrogen bonding and van der Waals forces. The binding distance *r* between the drugs and HSA were calculated according to the Forster non-radiative energy transfer theory. In this study, cordycepin was isolated for the first time from CP, and the interaction mechanisms between cordycepin and HSA were also studied for the first time.

CP has been commonly used as a Traditional Chinese Medicine [44]. Cordycepin, a compound with broad significant pharmacological functions, is known to be produced by 12 *Cordyceps* species [34] and *Aspergillus nidulans* [45]. CP is a good new source of cordycepin after *Cordyceps militaris*. The isolation of cordycepin from CP will help in research and establishment of the multiple pharmalogical attributes of this Traditional Chinese Medicine.

HSA is a very important protein in the plasma, which can transport, distribute, store and metabolize many drugs. The studies on the interaction between HSA and drugs can offer information of drug action to understand its distribution and absorption [46–48]. Cordycepin was used in clinical research against leukemia (*ClinicalTrials*.*gov*, verified by OncoVista, Inc., 2009), but there was no information about the effect of cordycepin on the biological activity of HSA. In this study, we first studied the interaction mechanisms between cordycepin and HSA, that may provide a substantial theoretical basis for full elucidation of the molecular mechanism of cordycepin transport and help the clinical application of cordycepin.

Cui *et al*. [28] reported the interaction between HEA and HSA with synthesized HEA. The mechanism of quenching fluorescence was not studied. In our study, we used natural HEA from CP to study the interaction of HEA and HSA. In addition, we studied the mechanism of quenching fluorescence. Cui *et al*. suggested that the interacting forces between HEA and HSA were hydrophobic and hydrogen bonding, but in our study they were hydrogen bonding and van der Waals forces. One reason for this difference may be the different sources of HEA and HSA used. Another may be different concentration of HSA and HEA used in fluorescence experiment. In our study, 0.5 μM HSA and 10, 20, 30, 40, 50 μM HEA were used, and that of Cui *et al*. were 0.4 μM HSA and 4.5, 9, 13.5, 18, 22.5 μM HEA.

In this study, cordycepin was isolated for the first time from CP, which will provide a good new source of cordycepin after *Cordyceps militaris* and enhance the use of this taxon. The interaction mechanisms between cordycepin and HSA may provide a substantial theoretical basis for elucidation of the molecular mechanism of cordycepin transport and help the clinical application of cordycepin. The interaction mechanisms between HEA and HSA is a good supplement for previous studies and a useful supplement for the clinical use of HEA.
